# Low utilization of health care services following screening for hypertension in Dar es Salaam (Tanzania): a prospective population-based study

**DOI:** 10.1186/1471-2458-8-407

**Published:** 2008-12-16

**Authors:** Pascal Bovet, Jean-Pierre Gervasoni, Mashombo Mkamba, Marianna Balampama, Christian Lengeler, Fred Paccaud

**Affiliations:** 1 University Institute of Social and Preventive Medicine (IUMSP), University Hospital Center and University of Lausanne, rue du Bugnon 17, 1005 Lausanne, Switzerland; 2 Temeke Municipal Medical Office of Health, Dar es Salaam, Tanzania; 3 Swiss Tropical Institute, P.O. Box 4002, Basel, Switzerland

## Abstract

**Background:**

Drug therapy in high-risk individuals has been advocated as an important strategy to reduce cardiovascular disease in low income countries. We determined, in a low-income urban population, the proportion of persons who utilized health services after having been diagnosed as hypertensive and advised to seek health care for further hypertension management.

**Methods:**

A population-based survey of 9254 persons aged 25–64 years was conducted in Dar es Salaam. Among the 540 persons with high blood pressure (defined here as BP ≥ 160/95 mmHg) at the initial contact, 253 (47%) had high BP on a 4^th ^visit 45 days later. Among them, 208 were untreated and advised to attend health care in a health center of their choice for further management of their hypertension. One year later, 161 were seen again and asked about their use of health services during the interval.

**Results:**

Among the 161 hypertensive persons advised to seek health care, 34% reported to have attended a formal health care provider during the 12-month interval (63% public facility; 30% private; 7% both). Antihypertensive treatment was taken by 34% at some point of time (suggesting poor uptake of health services) and 3% at the end of the 12-month follow-up (suggesting poor long-term compliance). Health services utilization tended to be associated with older age, previous history of high BP, being overweight and non-smoking, but not with education or wealth. Lack of symptoms and cost of treatment were the reasons reported most often for not attending health care.

**Conclusion:**

Low utilization of health services after hypertension screening suggests a small impact of a patient-centered screen-and-treat strategy in this low-income population. These findings emphasize the need to identify and address barriers to health care utilization for non-communicable diseases in this setting and, indirectly, the importance of public health measures for primary prevention of these diseases.

## Background

Worldwide, 7.1 million deaths (12.8% of the total) and 64.3 million DALYs (4.4% of the total) were due to sub-optimal blood pressure (BP) in 2000, and two thirds of the disease burden attributable to hypertension already occurs in the developing world [1]. It was estimated that 333 million adults had hypertension in economically developed countries and 639 million in economically developing countries [2]. In Tanzania, the mortality rate from non-communicable disease at the age of 15–59 years was markedly higher than in the UK [3], and a substantial prevalence of high BP was found in the population [4], including in our study area [5].

The efficacy of BP lowering medications is well demonstrated [6,7] and treatment of high-risk individuals has been advocated as a major strategy to curb non-communicable diseases in all countries, including in developing countries [8,9]. However, managing hypertension -or elevated total cardiovascular risk- is challenging in low-income countries for a variety of reasons, including drug availability and costs, as well as inadequacy of health services for identification and management of non-communicable diseases and their risk factors [10,11]. Also, individuals who struggle with a broad range of day-to-day problems may discount the benefit of long-term treatments for silent conditions that do not immediately jeopardize their health.

Attendance of a health care provider ("uptake of health services") is a first step that high-risk individuals must comply with in order to possibly benefit from treatment. Furthermore, uptake of health services should be followed by continued use of such health services for long-term treatment of hypertension and other non-communicable diseases. Studies that have examined barriers to the use of health services for the management of non-communicable diseases in developing countries have often examined this issue from the perspective of established users of health services (i.e. patients already attending health services) [12]. We are not aware of other studies that have assessed the utilization of health services for the management of non-communicable conditions in low-income countries on the basis of a population-based study. Population-based studies are essential to estimate the actual impact of a screen-and-treat strategy at the population level in such settings [8].

In this study, we examined the 12-month utilization of health services for hypertension management upon screening for hypertension in a large population-based sample of adults of a large city in sub Saharan Africa. Our reliance on hypertension alone (rather than on total cardiovascular risk) recognizes that the diagnosis of hypertension and long-term management of high BP can be relatively inexpensive and feasible (in contrast to diagnosis and long-term management of other main cardiovascular risk factors such as diabetes or dyslipidemia) and that a hypertension-based screen-and-treat strategy can be a practical entry point for the management of non-communicable diseases in low-resource settings. The secondary endpoint of this study was the identification of factors associated with the utilization of health services for hypertension.

## Methods

Dar es Salaam, with approximately 4 million inhabitants, is the economic center of Tanzania (about 40 millions inhabitants). The United Republic of Tanzania is situated on the East Coast of Africa, south of Kenya and north of Mozambique. According to the World Bank, the GDP for Tanzania was among the lowest worldwide (744 international dollars – purchasing power parity- in 2006) and total health expenditure amounted to only US$ 11.3 per capita per year [13].

In Dar es Salaam, medical care for hypertension (and diabetes) is given mainly through specialized outpatient clinics in government hospitals and in a few large public health centers-hence not in all public health centers- as well as in a few private hospitals and clinics. In 2005, several anti-hypertensive medications could be found at fairly similar prices in government and selected private pharmacies. The minimal cost for a one-year treatment was, for examples, US$1.5 for bendrofluazide (2.5 mg/day), $15 for atenolol (50 mg/day), $60 for amlodipine (5 mg/day) and $120 for captopril (50 mg/day) [14]. In comparison, a laborer's average salary can amount to US$30–50 per month. The 2000/01 Tanzanian Household Budget Survey reports that the mean expenditure per capita in Dar es Salaam was ~22 US$ per month, 2.9% of which went to medical expenditure.

Methods of the baseline survey, participants' characteristics and the distribution of BP and other risk factors in this population have been reported previously [5,15]. Briefly, eligible participants were all inhabitants aged 25–64 years living in 5 contiguous branches of the Temeke municipality of Dar es Salaam, in a low-income area of the city. The name and address of the inhabitants were available from a census performed one year before the study took place, as part of another health research program [3,16]. All examinations for this study took place at the participants' homes. Seventeen clinical officers, who had been trained for the conduct of the survey, made up to 4 attempts to meet the eligible participants at their home. In addition, the clinical officers could benefit from the help of the "ten-cell leaders" of the area (i.e. the persons administratively in charge of areas of around 10 adjacent houses or 100–200 people) for locating eligible participants and facilitating contacts.

**Figure 1:**
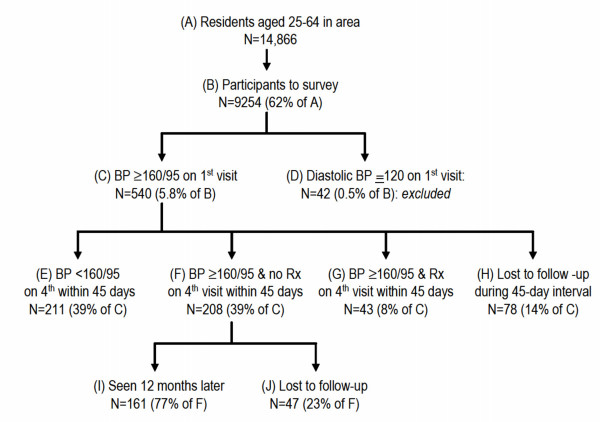
Study design (Rx: antihypertensive treatment).

Structured questionnaires were administered to the participants twice: i) at baseline("B" in Figure 1) and ii) at a follow-up visit 12 months after the participants had been screened for high BP and advised to seek health care for further management of hypertension ("I"). The baseline questionnaire inquired on socio-economic variables; lifestyle factors; history of hypertension and diabetes; knowledge, attitudes and practices related to cardiovascular risk factors and diseases; and various other characteristics. A wealth score (0 to 5) was constructed by adding 1 point for the availability, at the participants' home, of any of the following items: electricity (reported by 62% of inhabitants), television (26%), refrigerator (29%), flush toilet (15%) and private car (6%) [5]. Weight and height were measured with portable instruments. Blood was not collected and blood markers could not be assessed.

Humeral BP was measured with a validated automatic device (Visomat 2, Hestia Pharma, Mannheim, Germany) [17] with subjects in a sitting position. Three readings were obtained at each visit. The first reading was measured after a 5-minute rest and two subsequent readings were taken at intervals of ≥ 2 minutes. "High BP" was defined as systolic BP ≥ 160 mmHg and/or diastolic BP ≥ 95 mmHg based on the average of the last two of three readings. All persons who had high BP on the 1^st ^visit ("C" in Figure) had BP measured on 3 additional visits within 45 days. The participants who had high BP on the 4^th ^visit were considered as having "hypertension". This high BP cut off (≥ 160/95 mmHg) was chosen to maximize the specificity of the "hypertension" diagnosis, to limit the number of home visits during the 45-day period needed to ascertain hypertension, and to ensure that detected cases also had elevated total cardiovascular risk.

No intervention was provided until hypertension was ascertained on the 4^th ^visit. However, persons with diastolic BP ≥ 220 mm hg and/or diastolic BP ≥ 120 mmHg on any visit were advised to immediately report to a health care facility and they were excluded from the study. On the 4^th ^visit, all participants were informed of their hypertension status. All participants received a 10-minute advice on healthy lifestyles and nutrition and their relevance for BP control. In addition, persons with hypertension were told that i) they had hypertension, ii) they would most probably need to take antihypertensive medication on a long-term basis and iii) they should seek further health care in a health center of their choice for further management of their hypertension. In addition, the survey officers gave to all hypertensive participants a card with the participant's BP values on all 4 visits and a written advice to report to a health care facility within the next days. All survey officers were "clinical officers", i.e. health professionals who have completed a 2-year medical training program and who constitute a main resource for providing health care in government health centers in Tanzania (including prescribing essential medications, such as antihypertensive drugs).

All hypertensive participants who were not taking an antihypertensive medication at baseline were administered a follow-up questionnaire approximately 12 months later to assess health services utilization during the 12-month interval.

All participants were free to participate and verbal informed consent was sought from each participant. The study was approved by the National Institute of Medical Research and by the Tanzanian Commission for Science and Technology.

The main outcome of the study was the proportion of untreated hypertensive participants who attended a health care facility for the management of hypertension during the 12-month interval after hypertension screening and advice to seek health care. A secondary objective was to identify factors associated with health care utilization. Logistic regression was performed with minimal adjustment (age and sex) in view of the small sample size available for this analysis. Analyses were performed using Stata 8.2 and data are presented with 95% confidence intervals.

## Results

Participation is illustrated in Figure 1. Among the 9254 participants screened in the initial examination, high BP (defined as systolic/diastolic BP ≥ 160/95 mmHg in this study) was found in 582 individuals (6.3%) ("C"+"D" in Figure 1). Hypertension (defined as BP ≥ 160/95 mmHg on the 4^th ^visit in our study) was confirmed in 251 participants, i.e. in 47% of those with elevated BP on the first visit ("F"+"G"). Among the 208 participants with hypertension and who were not taking an antihypertension medication at baseline ("F"), we could trace 161 (77%) of them twelve months later ("I").

Table 1 shows that only 54 (34%) of the 161 hypertensive participants who were advised to seek health care actually attended a health care facility at least once during the 12-month follow-up period. Among them, 63% attended a public health care facility, 30% a private health care facility and 7% both.

**Table 1:** Factors associated with utilization of health services for hypertension treatment after hypertension screening and advice to seek health care for hypertension management

	Total with hypertension	Attended a health facility		Univariate association			Adjusted for age and sex		
				
	N	%	CI	OR	CI	*P*	OR	CI	*P*
Total	161	33.5	(26–49)						
Sex									
Men	60	28.3	(17–40)	1			1		
Women	101	36.6	(27–46)	1.46	(0.7–2.9)	ns	1.55	(0.8–3.1)	ns
Age									
25–44	63	23.8	(13–35)	1			1		
45–64	98	39.7	(30–50)	2.11	(1.1–4.3)	0.038	2.19	(1.1–.4.5)	0.031
Education									
None	53	39.6	(26–52)	1			1		
Primary	81	30.8	(21–41)	0.72	(0.3–1.4)	ns	0.93	(0.4–2.0)	ns
Secondary	27	29.6	'(11–48)	0.64	(0.2–1.7)	ns	0.81	(0.3–2.3)	ns
Wealth score									
0	76	34.2	(23–45)	1			1		
1–2	56	32.1	(20–45)	0.91	(0.5–1.8)	ns	1.08	(0.5–2.3)	ns
3–5	29	34.5	(16–53)	1.01	(0.4–2.5)	ns	1.08	(0.4–2.7)	ns
History of HBP									
No	90	27.8	(18–37)	1			1		
Yes	71	40.8	(29–52)	1.79	(0.9–2.3)	0.081	1.53	(0.8–3.0)	ns
Blood pressure									
< 180/110	119	33.6	(25–42)	1			1		
≥ 180/110	42	33.3	(19–48)	0.98	(0.5–2.0)	ns	0.83	(0.4–1.8)	ns
Diabetes									
No	157	33.7	(26–41)	1			1		
Yes	4	25.0	(0–100)	1.50	(0.2–15.0)	ns	2.19	(0.2–22)	ns
Overweight									
No	62	24.1	(13–32)	1			1		
Yes	69	39.2	(30–49)	2.10	(1.1–4.3)	0.038	1.99	(1.0–4.2)	0.069
Smoking									
No	142	35.9	(28–43)	1			1		
Yes	19	15.7	(0–33)	0.33	(0.1–1.2)	0.094	0.34	(0.1–1–3)	ns

OR: odds ratio; CI: 95% confidence interval.

Table 1 also shows that health care utilization was associated with older age (odds ratio 2.11, p < 0.05), being overweight (OR 2.1, p < 0.038), and marginally with smoking (OR: 0.33 p < 0.094) and history of hypertension (OR 1.79, p < 0.081). There was a non-significant trend towards larger health service utilization by women compared to men. Health care utilization was not associated with education, wealth, and BP level. The magnitude of these associations was similar after adjustment for age and sex, but only the association with age remained statistically significant.

**Figure 2:**
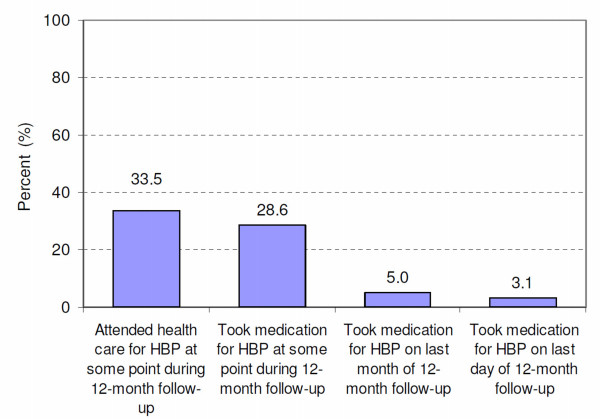
Proportion of all untreated hypertensive participants at baseline (n = 161) who attended health services for hypertension treatment and/or received antihypertensive treatment after hypertension screening and advice to seek health care for hypertension management.

Among the 161 hypertensive participants who could be traced, 46 (29%) reported to have been prescribed some antihypertensive treatment at some point during the 12-month interval, 8 (5%) during the last month and 5 (3%) on the last day (Figure 2). Among the 54 hypertensive persons who visited a health care facility, as advised, 85% took an antihypertensive treatment at some point during the 12-month interval, but only 14% during the last month and 9% on the last day. The proportion of participants taking antihypertensive medication on the last month/day may represent the proportions of hypertensive patients that are compliant over the long-term. The difference in BP before and after follow-up was not statistically different in treated vs. non-treated participants, which was anticipated in view of the small sample sizes.

Other results worth noting were found among the 161 hypertensive persons. Only 1 person reported to have sought health care at a traditional practitioner and 6 at a pharmacy (all of them also sought treatment at a regular health care provider). Forty-seven percent reported that hypertension was "very important" for them, 23% "moderately important" and the remaining 30% "not important". Asked about possible complications of hypertension along an open-ended question, 89 (55%) could not tell any but 29% mentioned stroke or paralysis and another 9% reported death. To the question on how long antihypertensive treatment should generally be taken, 83% did not know. Among the 115 of 161 hypertensive persons who did not attend health services for hypertension, 40% reported that the main reason for not seeking health care was the lack of symptoms associated with raised BP and 15% mentioned the cost of health care and treatment.

## Discussion

This study shows that, in a fairly poor area of an urban population of a low-income country, only one third of hypertensive persons advised to seek health care upon hypertension screening actually attended health services at any time during a 12-month follow-up interval. Furthermore, among those individuals who sought health care, less than 10% were taking antihypertensive medication at the end of the 12-month interval. These figures give an idea of both the uptake of health services upon screening of hypertension and the level of compliance with long-term treatment. Overall, these findings may indicate that the effectiveness of a community-based screen-and-treat strategy is likely to be low in this setting.

Despite several limitations (see below), this study raises at least two important points. The first is the way diagnosis of hypertension should be made. The two-fold decrease of the prevalence of high BP between the first and fourth visits stresses again the need to measure BP several times on each visit of several visits before the diagnosis of hypertension can be made. This allows to take into account the variability of BP measures and the progressive habituation of patients to BP measurement over time [15,18]. Identifying false positive cases using repeated BP readings is important everywhere to avoid unnecessary treatment, but this is even more crucial in resource-limited settings. This comment on BP measurements also applies for strategies based on an individual's total cardiovascular risk [9].

The second point is related to the low utilization of health services among persons identified as hypertensive after a careful diagnosis and subsequent advice to seek appropriate health care. Not only was the proportion of hypertensive persons who actually attended health care at least once low (one third), but an even smaller fraction (5%) reported using an antihypertensive medication one year later (suggesting a very low level of long-term compliance). Low utilization of health care for hypertension in our study is consistent with findings gathered in a study conducted at health service level in the same area (municipality of Temeke; population of approximately 1 million): among all 87,773 medical visits to all public and private health services during a one-month period, only 817 visits were related to hypertension (and 440 to diabetes) [14]. Our findings of a low utilization of health services for hypertension treatment are also consistent with very low proportions with BP controlled (BP < 140/90 mmHg) among hypertensives treated in population-based cross-sectional studies in many developing countries [4,19]. This proportion was as low as 2.6% in men and 4.3% in women in the population of this study [5].

Attendance of health care is a necessary first step to get appropriate health care for hypertension treatment. Low uptake of health services for the management of hypertension may reflect barriers related to the individuals (e.g., lack of awareness on hypertension, low priority given to asymptomatic conditions, competing priorities, costs, cultural factors, etc); health personnel (e.g. skills to adequately treat non-communicable diseases and underlying training); health systems (e.g. inadequate structures, inefficient organization, insufficient resources for health care of chronic conditions); and, more generally, a restricted focus on BP lowering programs and health policies in countries facing a double burden of communicable and non-communicable diseases [10,11]. Of note, the observations in this study correspond to a situation in which some organized health care exists for the diagnosis and treatment of hypertension (e.g. hypertension clinics in several hospitals and in some large health centers) and where anti-hypertensive medications can be purchased at all times (both in public and private pharmacies). This contrasts with many rural areas where such services are not available.

The marked decrease of persons under antihypertensive treatment over the 12-month follow-up interval points to poor compliance to long-term medication for an asymptomatic condition. However, a low proportion of patients under treatment at the end of the 12-month interval is not necessarily equivalent to poor adherence to treatment by the individuals. Patients may not be advised by their doctor (or nurse or clinical officer) to take a medication on a long-term basis. Low long-term use of antihypertensive treatment is also consistent with a common belief by the patients that hypertension can be treated with medications given for a few days only (as found in our study). Similarly, it is not unusual that hypertensive patients are treated with bed rest and short term medication (e.g. i.v. furosemide) only or antihypertensive medications are prescribed for just a few days or weeks. The cost of medications also remains a problem. Except for bendrofluazide (~US$1.5 for 12-month treatment), antihypertensive medicines were expensive in comparison with average wages [14]. However, in a study conducted in a middle-income country in the African region, good adherence to a simple one-pill antihypertensive regimen was only 26% one year after treatment was started, despite the fact that health care and medications were provided without direct fees to the patients [20].

Although this study was not primarily designed to identify factors associated with this poor performance, our data suggest that older age and past history of high BP could be predictive factors for health care utilization, consistent with other reports [21]. The lack of an association with education or wealth was unexpected. However it must be emphasized that the upper education and/or wealth categories in this poor area actually correspond to fairly low absolute levels. Findings could have been different if the range of education and wealth categories had been larger in the population. We found a trend toward lower health care utilization for hypertension treatment among smokers than non-smokers. We could speculate that this may reflect lower concern for health among smokers than non-smokers. An inverse relationship between health care utilization and smoking would be a matter of concern since smoking substantially increases the risk of cardiovascular disease and hypertensive smokers would therefore most benefit from antihypertensive treatment.

The study has several limitations. First, the stringent definition of hypertension in this study underestimated the true proportion of hypertension in the population (the proportion of persons with BP ≥ 140/90 mmHg in this population is described elsewhere [5]). However, the high cut off for hypertension used in this study (close to "grade 2" hypertension according to current guidelines [18]), ensured, on one hand, that most cases were truly hypertensive and, on the other hand, that most hypertensive cases were at increased total cardiovascular risk irrespective of other risk factors and therefore eligible for treatment. Most guidelines – including for the African region [22] – indeed recommend medication therapy in case of either high total cardiovascular risk or very high BP alone. Admittedly, further studies should examine the utilization of health services based on standard cut-off values for hypertension and using an explicit total risk approach to treatment. Second, we excluded participants under antihypertensive treatment at baseline. However, including them would not have markedly changed our conclusions since only few participants were taking antihypertensive treatment at baseline and some of them also might have dropped treatment during the one-year follow-up. Third, the participation rates at both the baseline and follow-up examinations seemed suboptimal. However, a characteristic of this poor urban study setting is that the population is rapidly changing, which is complicating the concept of the resident population. A sub-study in this area showed that, during a one-year period, 21% had moved outside of the area, 4.8% were traveling (sometimes for months) to their village or to take up some occasional work elsewhere, 3.8% had died, and the fate of 4.2% was unknown [5]. Hence, it is likely that our follow-up study has included most of the adults continuously living in the area during the 12-month follow up interval. Fourth, one may question the generalization of a screening program performed at home vs. within usual health service premises. It is however likely that the location of the screening at home had less influence than other factors that can impact on individuals' behaviors to actually seek further health care or not (cost, expected benefit of treatment, competing priorities, etc). Finally, with regards to our secondary aim of identifying correlates of health services utilization, we ended up with a fairly small number of hypertensive persons who attended a health facility for hypertension management (n = 161) despite having screened a large population (n = 9254), which limited the statistical power of these association analyses.

## Conclusion

In conclusion, our data suggest that the low uptake of health services for hypertension management and poor compliance to long-term treatment strongly limit the impact of a patient-centered screen-and-treat strategy in a low-income population. The current shift of focus in recent guidelines from a treatment approach based on single risk factors (such as hypertension in our study) to a treatment approach based on total cardiovascular risk [8,9] does not lessen the meaning of our findings and conclusions, since the utilization of health services is a necessary step in all instances. Furthermore, screening and treatment of hypertension may well be a most feasible and affordable therapeutic approach for increased cardiovascular risk in this setting. Low utilization of health services for hypertension treatment (and likely for the management of other chronic non-communicable conditions) is an important message for those in charge of the implementation of high-risk strategies in developing countries, as recently described in a series of seminal papers on non-communicable diseases in low income countries [8] and elsewhere [9]. Our study also emphasizes the need to identify and address barriers to effective delivery of health care for non-communicable diseases in low-resource settings [23]. The example of Cuba, another low-income country, provides some encouraging clues: hypertension control rates are among the highest worldwide [24], likely a response to a strong primary health care system and the local production of antihypertensive medications, among other factors. More generally, the suboptimal impact of a patient-centered treatment strategy further stresses the need to develop, experiment, and implement public health measures targeting the entire population for primary prevention of non-communicable diseases [25].

## Competing interests

The authors declare that they have no competing interests.

## Authors' contributions

PB designed the study, analyzed the data analysis and drafted the paper. JPG designed the study and participated to data analysis and write up. MM and MB coordinated the conduct of the study and reviewed the paper. CL and FP contributed to the study design and reviewed the paper.

## Pre-publication history

The pre-publication history for this paper can be accessed here:


